# Vitamin C intake potentially lowers total cholesterol to improve endothelial function in diabetic patients at increased risk of cardiovascular disease: A systematic review of randomized controlled trials

**DOI:** 10.3389/fnut.2022.1011002

**Published:** 2022-10-31

**Authors:** Phiwayinkosi V. Dludla, Bongani B. Nkambule, Tawanda M. Nyambuya, Khanyisani Ziqubu, Sihle E. Mabhida, Vuyolwethu Mxinwa, Kabelo Mokgalaboni, Fransina Ndevahoma, Sidney Hanser, Sithandiwe E. Mazibuko-Mbeje, Albertus K. Basson, Jacopo Sabbatinelli, Luca Tiano

**Affiliations:** ^1^Biomedical Research and Innovation Platform, South African Medical Research Council, Tygerberg, South Africa; ^2^Department of Biochemistry and Microbiology, University of Zululand, KwaDlangezwa, South Africa; ^3^School of Laboratory Medicine and Medical Sciences, University of KwaZulu-Natal, Durban, South Africa; ^4^Department of Health Sciences, Namibia University of Science and Technology, Windhoek, Namibia; ^5^Department of Biochemistry, North-West University, Mmabatho, South Africa; ^6^Department of Life and Consumer Sciences, University of South Africa, Florida Campus, Roodepoort, South Africa; ^7^Department of Physiology and Environmental Health, University of Limpopo, Sovenga, South Africa; ^8^Department of Clinical and Molecular Sciences, Polytechnic University of Marche, Ancona, Italy; ^9^Department of Life and Environmental Sciences, Polytechnic University of Marche, Ancona, Italy

**Keywords:** vitamin C, dietary supplements, antioxidants, diabetes mellitus, metabolic syndrome, cardiovascular diseases

## Abstract

**Background:**

Vitamin C is one of the most consumed dietary compounds and contains abundant antioxidant properties that could be essential in improving metabolic function. Thus, the current systematic review analyzed evidence on the beneficial effects of vitamin C intake on cardiovascular disease (CVD)-related outcomes in patients with diabetes or metabolic syndrome.

**Methods:**

To identify relevant randomized control trials (RCTs), a systematic search was run using prominent search engines like PubMed and Google Scholar, from beginning up to March 2022. The modified Black and Downs checklist was used to assess the quality of evidence.

**Results:**

Findings summarized in the current review favor the beneficial effects of vitamin C intake on improving basic metabolic parameters and lowering total cholesterol levels to reduce CVD-risk in subjects with type 2 diabetes or related metabolic diseases. Moreover, vitamin C intake could also reduce the predominant markers of inflammation and oxidative stress like C-reactive protein, interleukin-6, and malondialdehyde. Importantly, these positive outcomes were consistent with improved endothelial function or increased blood flow in these subjects. Predominantly effective doses were 1,000 mg/daily for 4 weeks up to 12 months. The included RCTs presented with the high quality of evidence.

**Conclusion:**

Clinical evidence on the beneficial effects of vitamin C intake or its impact on improving prominent markers of inflammation and oxidative stress in patients with diabetes is still limited. Thus, more RCTs are required to solidify these findings, which is essential to better manage diabetic patients at increased risk of developing CVD.

## Introduction

**FIGURE 1:**
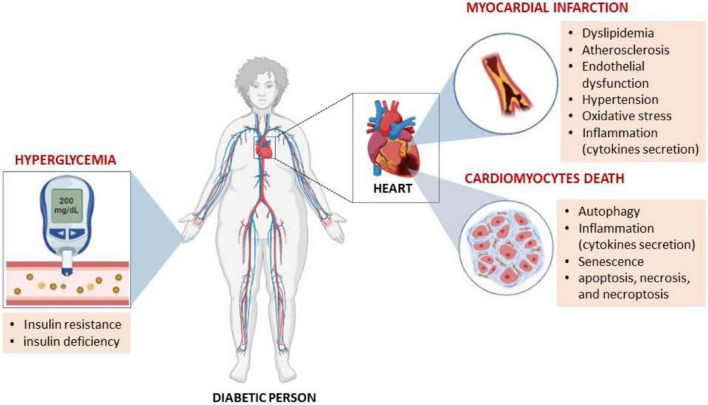
Hyperglycemia, as a major characteristic feature of diabetes mellitus, is known be responsible for prompting metabolic abnormalities that make an individual susceptible to increased cardiovascular disease (CVD)-risk. For example, abnormal levels of serum lipids (dyslipidemia) that may eventually cause atherosclerosis through endothelial dysfunction are all implicated in the development of CVDs. Similarly, preclinical studies have implicated pathological mechanisms such as abnormal autophagy and inflammation to be involved in this process.

Diabetes mellitus remains one of the leading causes of deaths worldwide (1). Although currently used antidiabetic therapies such as metformin and insulin can manage diabetes-associated complications (2, 3), their long-term therapeutic effects could be limited due to the rapid rise of diabetes-related deaths (4). Certainly, most diabetic patients (mainly due to pathological consequences of hyperglycemia) are known to be at increased risk for cardiovascular disease (CVD)-related deaths (Figure 1) (5, 6). Thus, in addition to understanding the precise pathological mechanisms implicated in diabetes-induced myocardial injury, there has been a growing need to discover novel pharmacological compounds, with strong cardioprotective properties to prolong the lives of diabetic patients.

There has been a general interest in pharmacological compounds with strong antioxidant and anti-inflammatory properties for their protective effects against diabetes-associated cardiovascular complications (7–12). This is important since impaired glucose intolerance in a diabetic state has been associated with aggravated pro-inflammatory response and oxidative stress-induced vascular damage (13, 14). Consistently, due to their perceived ameliorative effects against inflammation and oxidative stress, there has been a great necessity to examine the protective effects of dietary compounds against diabetes-associated complications (15–17).

There is currently a considerable interest in understanding the therapeutic role of herbs and supplements against diabetes and CVD-related complications (18–20). This includes uncovering the therapeutic effects of vitamins, which are considered vital micronutrient that an organism requires for an adequate metabolic function. Indeed, various dietary compounds such as vitamin C are increasingly consumed for their envisaged benefits against metabolic complications (21–23). Vitamin C is considered an essential nutrient that functions as a vital antioxidant in protecting against oxidative stress and tissue damage (24, 25). Besides its availability as a dietary supplement, vitamin C can also be found in food sources including citrus fruits and vegetables. The beneficial effects of vitamin C are associated with their capacity to attenuate oxidative stress and inflammation (21). A previously published meta-analysis showed that vitamin C intake could improve glycemic control or blood pressure in adult participants (22, 26). More evidence is required to understand the health benefits of this dietary antioxidant, and to potentially curb the rising toll of CVD-related deaths in patients with diabetes or metabolic diseases. This is in support of recent reviews highlighting the gap in clinical evidence informing on the favorable outcomes of vitamin C in individuals at increased CVD-risk (27–29). Importantly, although other reviews have reported on the potential therapeutic effects of vitamin C on controlling basic metabolic function and CVD-related outcomes (26, 30, 31), very limited information exists on the implications or link with biomarkers of inflammation and oxidative stress such as C-reactive protein (CRP) and interleukin 6 (IL-6), and malondialdehyde (MDA) levels.

### Methodology

Supplementary file 1 contains the Preferred Reporting Items for Systematic reviews and Meta-Analysis (PRISMA) guidelines that were followed to prepare the manuscript. The current study does not have an approved protocol; however, the International prospective register of systematic reviews (PROSPERO) was cautiously surveyed to avoid duplicating systematic reviews or meta-analysis investigating a similar topic.

### Approach for searching randomized controlled trials

Briefly, a systematic search was run using prominent search engines like PubMed and Google Scholar, from beginning up to March 2022. This was done by two independent reviewers. To optimally cover relevant literature, a rather broad primary search strategy was applied where we explored all randomized controlled trials (RCTs) reporting on vitamin C intake in patients with diabetes or metabolic syndrome. Thereafter, the especial focus was placed on RCTs assessing the effect of vitamin C intake on outcomes related with CVD in diabetic individuals, and this was done in comparison to the placebo or comparative control. Medical Subject-Heading (MeSH) and text words “vitamin C,” “diabetes,” “metabolic syndrome,” “cardiovascular disease,” and their matching synonyms were used.

### Study selection

The study encompassed RCTs reporting on the therapeutic effects of vitamin C on outcomes related with CVD in adults (>18 years) with diabetes or metabolic syndrome. Notably, encompassed RCTs were those that assessed the use of vitamin C as an intervention, comprising the comparison group on placebo, reporting on any quantifiable outcome of CVD in patients with diabetes or metabolic syndrome. RCTs reporting on the use of vitamin C in conjunction with other therapies were excluded. Also, RCTs not describing the clear CVD-outcome or covered incomplete information were excluded. Relevant items, including participants, interventions, comparisons, and outcomes (PICO), are described below:

• Participants:

Adult patients with diabetes and at increased risk of developing CVD.

• Interventions:

Treatment intervention involved vitamin C intake in patients with diabetes or metabolic syndrome.

• Comparisons:

Patients receiving placebo were used a comparative control.

• Outcomes:

The primary outcomes for this systematic review included basic metabolic profiles such as blood glucose levels, and CVD-risk measurements like lipid profiles, endothelial function, and blood pressure. Whereas the secondary outcome were biomarkers of inflammation and oxidative stress.

### Data extraction and assessment of quality

Briefly, qualifying articles were carefully selected by at least two independent investigators. The main outcome of the study was to assess the effects of vitamin C intake on outcomes related with CVD in diabetes or condition of metabolic syndrome. It remained imperative to also assess correlation between duration of intervention and improvements in CVD-related outcomes in the study population. For accurate reporting, relevant data items extracted from each RCT included the name and year of publication, the country where the study was performed, sample and gender dissemination, as well as the dose and duration of intervention. The risk of bias was independently assessed by at least two investigators using of the adapted Downs and Black checklist, which is appropriate for both randomized and non-randomized studies (32, 33).

## Overview of study findings

### Study selection

**FIGURE 2:**
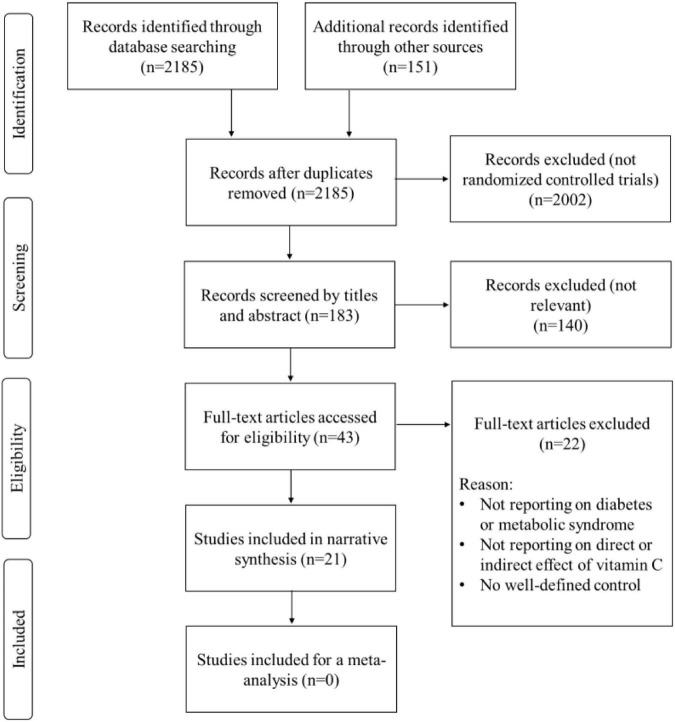
A summary of flow diagram presenting study selection.

A total of 183 RCTs were inspected for eligibility, however, only 21 studies were selected, as shown in Figure 2. All encompassed studies were RCTs on the effects of vitamin C intake on outcomes related with CVD in patients with diabetes or metabolic syndrome. Disqualified studies were on the combination use of vitamin C with other pharmacological compounds, or for not having a well-defined control group. Other exclusions were related for not having a clear study design and not reporting on the effect of vitamin C on the predefined study population.

### Study characteristics

All contained RCTs were available from peer-reviewed journals, as shown in Table 1. Besides Australia (*n* = 3), Iran (*n* = 2), New Zealand (*n* = 1), Palestine (*n* = 1), most studies were from the United States (*n* = 4), United Kingdom (*n* = 5), and Europe (*n* = 5). Overall, the total of participants was 7,688, with an average age of 60 years, with at least 50% of them registered as males (Table 1). Furthermore, approximately 90% of RCTs evaluated type 2 diabetes (T2D), while the rest were focused on patients with type 1 diabetes and the metabolic syndrome (Table 1). In terms of dose selections, consistency was observed where most studies used vitamin C at 1,000 mg, taken once a day, or twice daily at doses of 500 mg (Table 1). The treatment duration was consistent at 4–6 weeks (Table 1), while other RCTs did evaluate short term effects of vitamin C intake in terms of hours a few days (34–36), whereas limited studies tested the long-term effects at 4 months (37–39), 1 year (40), 4 years (41), and 9.2 years (42).

**TABLE 1:** An overview of studies reporting on the impact of vitamin C intake on cardiovascular disease (CVD)-related outcomes.

Study	Country	Study size	Male, (%)	Age (years)	Vitamin C dose and duration (g/mg)	Main findings
Klein et al. (50)	Denmark	23 patients with type 1 diabetes (T1D)	100	32.5	Vitamin C at 500 mg twice a day, being daily doses of 6 g for 4 weeks	Vitamin C did not affect renal hemodynamics
Paolisso et al. (37)	Italy	40 patients with type 2 diabetes (T2D)	48	72 ± 0.5	Vitamin C at 0.5 g twice daily for 4 months	Vitamin C improved whole body glucose disposal and non-oxidative glucose metabolism. While plasma vitamin C levels were consistent with a decrease in plasma low-density lipoprotein (LDL)-cholesterol and insulin levels. Treatment was also correlated to reduced plasma free radicals and increase in glutathione
Mayer-Davis et al. (41)	United States	520 Insulin Resistance Atherosclerosis Study (IRAS) and 422 San Luis Valley Diabetes Study (SLVDS)	56 IRAS, 57 SLVDS	57 IRAS, 59 SLVDS	Vitamin C intake at 275 or 133 mg daily IRAS and SLVDS, respectively for 4 years	Vitamin C intake did not affect systolic or diastolic blood pressure, including the levels of high-density lipoprotein (HDL) or LDL cholesterol, or triglycerides
McAuliffe et al. (40)	Australia	20 diabetic patients: 2 with T1D and 18 with T2D	75	58 ± 12	Vitamin C at 500 mg twice daily for 12 months	Vitamin C intake elevated its plasma levels and reduced albumin excretion rate
Upritchard et al. (53)	New Zealand	25 patients with T2D	64	58 ± 7.5	Vitamin C at 500 mg daily for 4 weeks	Vitamin C intake did not significantly affect fasting plasma glucose (FPG), LDL oxidation or C-reactive protein levels
Darko et al. (47)	United Kingdom	35 patients withT2D	65	56.1 ± 1.5	Vitamin C at 1.5 g daily in three doses for 3 weeks	Vitamin C intake did not markedly affect oxidative stress, blood pressure or endothelial function in patients with T2D
Mullan et al. (54)	United Kingdom	30 patients with T2D	73	59.4 ± 6.6	Vitamin C at 500 mg daily for 4 weeks	Vitamin C intake reduced brachial systolic and diastolic blood pressure, concurrent to improving arterial stiffness
Morel et al. (34)	France	61 patients with myocardial infarction (*n* = 23 was diabetic)	87	62 ± 9	Vitamin C at 1 g daily for 5 days	Vitamin C intake lowered platelet-derived microparticles. Treatment also reduced endothelial and platelet-derived microparticles
Tousoulis et al. (52)	Greece	39 patients with T2D and coronary artery disease	87	65.3 ± 1.6	Vitamin C at 2 g daily for 4 weeks	Vitamin C intake increased blood flow by elevating reactive hyperemia, and decreasing plasma tissue plasminogen activator and von Willebrand factor
Mullan et al. (35)	United Kingdom	12 healthy men subjected to acute hyperglycemia	100	25.2 ± 4.1	Vitamin C at 2 g initiated before hyperglycemia	Vitamin C pre-treatment prevented the hyperglycemia-induced hemodynamic changes, including brachial systolic or diastolic pressure
Lu et al. (43)	Sweden	17 patients with T2D	71	54	Vitamin C at 1 g three times a day for 2 weeks	Vitamin C intake did not affect microvascular reactivity evaluated at the level of individual capillaries. Furthermore, this compound did influence inflammatory cytokines or oxidized LDL
Anderson et al. (46)	United Kingdom	20 patients with T2D	70	53.2 ± 7.4	Vitamin C at 1 g for 2 days prior to re-testing and with the fat meal	Vitamin C intake enhanced endothelial function at all time points and diminished post-prandial lipemia-induced oxidative stress
Chen et al. (45)	United States	32 patients with T2D	42	50 ± 1	Vitamin C (800 mg/day) for 4 weeks	Vitamin C plasma increased but did not affect FPG, insulin, or forearm blood flow in response to acetylcholinesterase (ACh)
Afkhami-Ardekani et al. (48)	Iran	84 patients with T2D	51	52.3 ± 9.6	Vitamin C at 500 mg or 1,000 mg daily for 6 weeks	Vitamin C intake reduced FPG, triglycerides, LDL, glycated hemoglobin (HbA1c) and serum insulin (at a dose of 1,000 mg). The lower dose did not have an effect
Davison et al. (36)	United Kingdom	12 patients with T1D	100	27 ± 3.5	Vitamin C bolus of 1 g 2 h prior to the exercise challenge	Vitamin C intake elevated its plasma concentration to a similar degree in both groups and reduced the exercise-induced oxidative stress response
Song et al. (42)	United States	6,574 patients with T2D	0	60.8 ± 8.9	Vitamin C at 500 mg every day for 9.2 years	Vitamin C intake was associated with a trend toward a modest reduction in diabetes risk in women compared to placebo
Mazloom et al. (51)	Iran	27 patients with T2D	30	47 ± 8.2	Vitamin C at 1,000 mg daily for 6 weeks	Vitamin C intake decreased FPG and malondialdehyde (MDA) levels when compared to placebo. But it did not affect lipid profiles
Gutierrez et al. (49)	United States	8 patients with T2D	50	49 ± 6	Vitamin C at (250/500/1,000 mg daily for 2 weeks	Vitamin C elevated its concentrations at all dosages. However, no significant effect was seen on lipid parameters or any of the markers of oxidative stress, inflammation, or hypercoagulability
Ellulu et al. (44)	Palestine	64 patients with metabolic syndrome	31	50.7 ± 8.5	Vitamin C at 1 g (500 mg twice per day) for 8 weeks	Vitamin C intake decreased the levels of high-sensitivity C-reactive protein, interleukin 6 (IL-6), FBG, and triglycerides. But did not affect total cholesterol
Mason et al. (38)	Australia	14 patients with T2D	86	59.4 ± 3.5	Vitamin C at 500 mg twice daily for 4 months	Vitamin C intake enhanced insulin-mediated glucose disposal, peripheral insulin-sensitivity index, including its skeletal muscle concentration and muscle sodium-dependent vitamin C transporter 2 protein expression. It further decreased skeletal muscle reactive oxygen species (ROS) production. Total superoxide dismutase (SOD) activity was also reduced. But did not affect basal oxidative stress markers, citrate synthase activity, endogenous glucose production, HbA1c and muscle protein kinase B expression
Mason et al. (39)	Australia	31 patients with T2D	84	61.8 ± 6.8	Vitamin C at 500 mg twice daily for 4 months	Vitamin C intake decreased FPG, as well as systolic and diastolic blood pressures

### Risk of bias assessment

Briefly, the modified Back and Downs checklist with 26 questions and four domains, which are relevant for analyzing the quality of encompassed studies (32). Out of the 21 included studies six were excellent (38, 39, 42–45), 12 were scored as good (35, 37, 40, 46–54), and three were fair (34, 36, 41). Supplementary file 2 depicts that encompassed studies presented with low reporting bias with a median score of 8 out of a probable score of 10 (overall agreement 85.67%, kappa = 0.71), good external legitimacy with median score of 2 out of 3 (overall agreement 39.13%, kappa = 0.13), excellent internal validity with median score 7 out of 7 (overall agreement 65.22%, kappa = 0.34) and low risk assortment bias with median of 5 out of possible 6 (overall agreement 56.51%, kappa = 0.19). Therefore, interpretation of the findings can be trusted and applied outside the selected study population.

### Evidence on the impact of vitamin C intake of metabolic and cardiovascular disease-related outcomes

The overall included studies reported on vitamin C intake and its diverse effects on basic metabolic parameters such as glycated hemoglobin (HbA1c), fasting plasma glucose and insulin levels, as well as CVD related outcomes including lipid profiles and blood pressure (Table 1).

In relation to basic metabolic parameters, evidence available from as early as 1995 indicated that intake of vitamin C, at 500 mg twice daily for 4 months, could improve whole body glucose disposal and non-oxidative glucose metabolism in individuals with T2D (37). Interestingly, this was consistent with enhanced plasma vitamin C levels, a decline in plasma low-density lipoprotein (LDL)-cholesterol, as well as reduced free radicals and insulin levels. McAuliffe (40), confirmed some of these findings showing that vitamin C intake at a similar dose (500 mg) twice daily for 12 months could increase its plasma levels while resulting in reduced albumin excretion rate in diabetic patients. As new information became available, Mullan et al. (54) demonstrated that vitamin C intake at 500 mg daily for 4 weeks reduced brachial systolic and diastolic blood pressure concomitant to improving arterial stiffness in patients with T2D. Whereas Morel et al. (34) showed that vitamin C intake at 1,000 mg daily for 5 days reduced platelet-derived microparticles in diabetic patients with myocardial infarction.

Other beneficial effects linked with vitamin C intake in patients with diabetes or metabolic syndrome extended to improving blood flow or lowering blood pressure (35, 38, 39, 52); attenuating oxidative stress and endothelial dysfunction (36, 38, 39, 46). This was consistent with an effective control of fasting plasma glucose (FPG), triglycerides, LDL, HbA1c and serum insulin levels (38, 39, 48, 51); while also reducing the pro-inflammatory markers such as CRP and IL-6 (44). These positive effects with vitamin C intake were predominantly observed with the doses of 1,000 mg/daily, and intervention period of 4 weeks up to 12 months in patients with T2D. Alternatively, in patients with T1D, vitamin C intake could enhance its plasma levels and these effects were linked with reduced oxidative stress response (36, 40). However, very few studies have investigated the therapeutic effects of vitamin C in patients with T1D.

Opposing the advantages observed with vitamin C intake in controlling metabolic disease associated complications in diabetic patients, other studies did not report any positive effects with regular intake of this antioxidant. For example, Klein et al. (50) showed that vitamin C at 500 mg twice a day for 4 weeks could not normalize renal hemodynamics in normoalbuminuric in normotensive diabetic patients. Mayer-Davis et al. (41) revealed that vitamin C intake at 275 or 133 mg daily for 4 years did not impact systolic or diastolic blood pressure nor with HDL or LDL cholesterol, or triglycerides. Similarly, Upritchard et al. (53) and Darko et al. (47) showed that vitamin C intake between the doses of 500 and 1,500 mg daily for 3–4 weeks did not significantly improve endothelial function or affect FPG, LDL oxidation, or C-reactive protein levels in patients with T2D. Consistently, other studies showed that regular intake of vitamin C at 800–1,000 mg for 2–4 weeks did not significantly affect inflammatory cytokines, oxidized LDL, FPG or insulin levels, or forearm blood flow in patients with T2D (43, 45, 49). Notably, except for an RCT by Mayer-Davis et al. (41) which used approximately 520 participants, most of the studies that did not observe any significant results used a very low sample number, indicating this could have affected the power of the results. However, this is only a hypothesis, well-designed RCTs with adequate sample number are still required to give a better picture on the impact of vitamin C on outcomes related with CVD in patients with diabetes or metabolic syndrome.

## Discussion

### A gap in available clinical evidence

The rapid prevalence of diabetes (55), coupled with the lack of effective therapies for its management (56), has propelled research into establishing alternative approaches to prevent this calamity. Dietary supplements have become an attractive target to investigate for their health benefits, especially due to their envisaged safety profile and potential bioactive properties (16, 57, 58). Vitamin C is considered an essential nutrient, also forming part of the World’s Health Organizations List of Essential Medicine, with abundant antioxidant effects (59, 60). Although many factors such as absorption and bioavailability profiles can influence its physiologic concentrations (61), it has long estimated that adequate intake of vitamin C of either 200 mg/day from five servings of fruits and vegetables or 100 mg/day is necessary to prevent its deficiency with a margin of safety (62). Consistently, epidemiological data support the observed improvements in CVD-related outcomes with high consumption of fruits and vegetables rich in antioxidants like vitamin C and E (63–66). Alternatively, while experimental data suggest vitamin C and E can protect against oxidative stress-induced cellular damage by scavenging of reactive oxygen species or by neutralizing lipid hydroperoxyl radicals (60), convincing evidence on the effect of multiple dietary supplements on metabolic and cardiovascular health is scarce (67). In fact, although quantitative analysis supports the positive effects of vitamin C intake on improving blood glucose control or blood pressure in patients with T2D (22, 26), such evidence has not been linked with CVD-related outcomes in conditions of metabolic syndrome.

### Summary of results supporting the beneficial effects of vitamin C

The current systematic review involved 21 RCTs, with 7,688 participants, evaluating the impact of vitamin C intake on basic metabolic parameters such as HbA1c, FPG, and insulin levels, as well as CVD-related outcomes including lipid profiles and blood pressure (Table 1). In fact, overwhelming studies supported the beneficial effects of vitamin C intake on improving metabolic function and reducing cholesterol levels in patients with diabetes or metabolic syndrome. In addition to reducing the pro-inflammatory markers such as CRP and IL-6 (44), vitamin C intake at an average dose of 1,000 mg daily between 3 weeks up to a period of 12 months showed consistent results lowering FPG, triglycerides, LDL, HbA1c, and serum insulin levels (38–40, 48, 51). Interestingly, these results were supported by significantly increased plasma levels of this antioxidant after its intake in these patients (Table 1). Notably, the reduction in total cholesterol was associated with attenuation of oxidative stress and amelioration of endothelial dysfunction in these patients (36, 38, 39, 46). Further suggesting that vitamin C may exerts its therapeutic effects by terminating oxidation of lipid products that are linked with exacerbation of inflammation and endothelial dysfunction, as demonstrated elsewhere (61, 68–70). This result could also translate to improve blood flow and reduced blood pressure, as demonstrated in some RCTs (35, 38, 39, 52). However, clinical evidence on the beneficial effects of vitamin C intake or its impact on improving prominent markers of inflammation and oxidative stress in patients with diabetes is still limited. Thus, more RCTs are required to solidify these findings, which is essential to better manage diabetic patients at increased risk of developing CVD.

### Summary of results not supporting the beneficial effects of vitamin C

Other studies included within the current systematic review reported that vitamin C intake did not influence markers of metabolic function or CVD. Notably, some studies showed that intake with vitamin C at an average dose of 1,000 mg daily for 2–4 weeks does not significantly affect blood pressure nor with HDL and LDL cholesterol, as well as triglycerides (41, 50). Even when taken at a lower dose of 275 or 133 mg daily for a prolonged period of 4 years (41), it was apparent this antioxidant does not affect systolic or diastolic blood pressure nor with HDL or LDL cholesterol, or triglycerides. Others showed that vitamin C intake at 800–1,000 mg for 2–4 weeks also does not impact inflammatory cytokines, oxidized LDL, FPG or insulin levels, or forearm blood flow in patients with T2D (43, 45, 49). These results are like others showing failure of vitamin C intake to improve metabolic disease-related complications or to protect against CVDs in various clinical settings (71, 72). However, this evidence is still limited, additional studies are required to confirm any of these findings. Preclinical studies can serve as the reference model to further explore other potential benefits of vitamin C. This is essential since various preclinical models have demonstrated that this antioxidant can ameliorate the pathological consequences of inflammation and oxidative stress to alleviate diabetes-associated complications (73–76).

## Conclusion and future perspective

**FIGURE 3:**
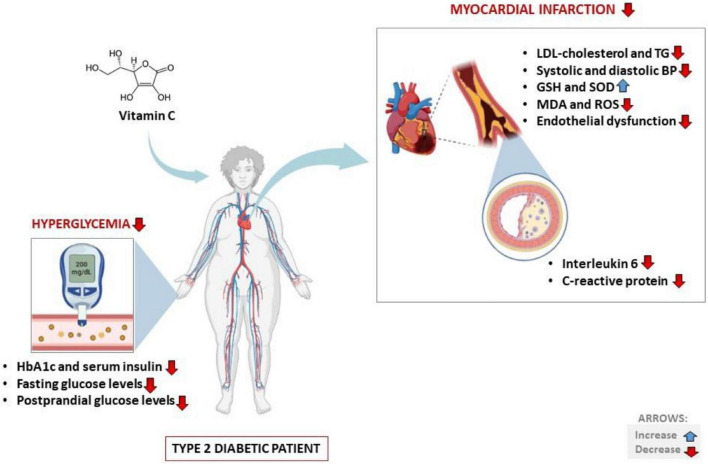
Potential health benefits of vitamin C intake on cardiovascular disease-related outcomes in type 2 diabetic patients. Briefly, besides improving basic metabolic parameters such as impaired glucose/insulin levels, vitamin C intake ca also lower total cholesterol concentrations to reduce blood pressure and positively affect blood circulation. HbA1c, glycated hemoglobin; LDL, low-density lipoprotein; TG, triglycerides; GSH, glutathione; SOD, superoxide dismutase; MDA, malondialdehyde; ROS, reactive oxygen species.

Summarized evidence showed that vitamin C intake could potentially improve basic metabolic profile while markedly reducing the levels of total cholesterol in patients with T2D and the metabolic syndrome (Figure 3). This was consistent with improved endothelial function, which is consistent to previous reports (35, 38, 39, 52). The fact that most antioxidant therapies have been dismissal in diabetes in clinical trials (77), highlights the need for additional RCTs to confirm these effects, especially for patients with T1D. Indeed, by searching the PubMed engine, very few RCTs could be accessed/identified reporting on the effects of vitamin C intake in patients with diabetes (78). Thus, additional clinical trials should consider the use of larger sample sizes and lengthier intake periods that are driven to stratify effects on the basis of baseline glycemic control necessary to validate favorable outcomes of vitamin C intake. Future considerations should include making use of a qualitative approach “meta-analysis” to strengthen the presented evidence. Which is one of the limitations of the current review, since most included RCTs were too diverse and presented very substantial heterogeneity and this could have affected the interpretation of results.

## Data availability statement

The original contributions presented in this study are included in the article/Supplementary material, further inquiries can be directed to the corresponding author.

## Author contributions

PD, BN, and LT: concept and original draft. PD, BN, and TN: literature search and data extraction. VM, KM, and FN: assess quality of evidence. All authors: writing and final approval of the manuscript.

## Supplementary material

Supplementary file 1 Preferred Reporting Items for Systematic reviews and Meta-Analysis (PRISMA) checklist.

Supplementary file 2 Downs and Black checklist.

## Abbreviations

CVD: cardiovascular disease
CRP: C reactive protein
DBP: diastolic blood pressure
FPG: fasting plasma glucose
IL: interleukin
GRADE: Grading of Recommendations Assessment Development and Evaluation
HbA1c: glycated hemoglobin
HDL: high-density lipoprotein
LDL: low-density lipoprotein
MDA: malondialdehyde
PRISMA: Preferred Reporting Items for Systematic reviews and Meta-Analysis
RCT: randomized controlled trials
SBP: systolic blood pressure
SMD: standard mean difference
T2D: type 2 diabetes.
